# Recurrent Idiopathic Spontaneous Pneumoperitoneum: A Case Report

**DOI:** 10.7759/cureus.26471

**Published:** 2022-06-30

**Authors:** David R Velez

**Affiliations:** 1 Surgery, University of North Dakota, Grand Forks, USA

**Keywords:** general surgery, surgery, conservative management, idiopathic spontaneous pneumoperitoneum, pneumoperitoneum

## Abstract

The most common cause of pneumoperitoneum is perforated hollow viscus, which generally necessitates emergent surgical intervention. Idiopathic spontaneous pneumoperitoneum (ISP) is a rare condition less commonly described. This report outlines the case of a 79-year-old male with recurrent idiopathic spontaneous pneumoperitoneum managed by repeated laparotomy. Knowledge of this rare phenomenon and appropriate workup may allow for the avoidance of unnecessary laparotomies. Despite this, definitively ruling out perforated hollow viscus is difficult outside of the operating room, and many patients will ultimately be taken for surgical exploration and definitive diagnosis.

## Introduction

Pneumoperitoneum is the presence of free air within the peritoneum. When seen on radiographic imaging, it is most commonly due to intra-abdominal emergencies such as perforated viscus [1]. Pneumoperitoneum, however, has numerous other possible etiologies that need to be considered. Idiopathic spontaneous pneumoperitoneum (ISP) is the presence of pneumoperitoneum with no clear etiology. Recurrent ISP is a rare disease and has only been described in a few case reports [1-3]. This report documents a case of recurrent ISP and reviews the presentation, diagnosis, and management of this rare condition.

## Case presentation

**Figure 1:**
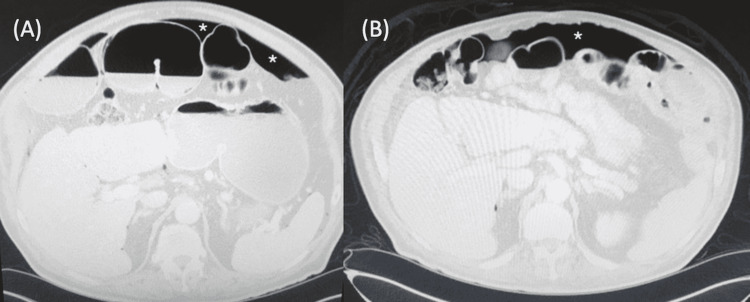
Pneumoperitoneum (asterisk) seen on CT from the patient’s first admission (A) and second admission (B).

A 79-year-old male presented to the emergency department with two days of nausea, vomiting, and abdominal pain. His past medical history was significant for coronary artery disease, hypertension, and diabetes. He had no past surgical history. He smoked one pack per day with no drug or alcohol use. On physical examination, his heart rate was in the low 100s but otherwise was hemodynamically stable. He had moderate abdominal distention but was nontender and otherwise unremarkable. Laboratory tests saw a mild leukocytosis of 12,100 cells per μL but were otherwise within normal limits. Computed tomography (CT) scan was the initial imaging and saw moderate pneumoperitoneum (Figure 1). He was therefore taken to the operating room for emergent diagnostic laparoscopy. After two hours of exploration, no pathology could be identified. The decision was then made to convert to an exploratory laparotomy. There was a small segment of proximal jejunum that had a questionable pneumatosis, but no real perforation or injury could be identified. Given that this was the only possible abnormality seen, the decision was made to resect this portion of the small bowel. He struggled with postoperative ileus, but this resolved on postoperative day 8, and he was discharged on postoperative day 11.

The patient returned to the emergency department two years later. He complained of nausea and vomiting for the past 12 hours but denied any abdominal pain. Vital signs remained stable. His physical examination again saw abdominal distention but was otherwise unremarkable. Laboratory tests again saw a mild leukocytosis of 14,700 cells per μL but were otherwise within normal limits. CT again saw moderate pneumoperitoneum (Figure 1). He was again taken emergently to the operating room for exploratory laparotomy. The entire small bowel was dilated, but there was no pneumatosis or any acute pathological findings despite a long and thorough exploration. His postoperative course was uncomplicated, and he was discharged on postoperative day 3.

## Discussion

ISP is a rare condition with only a few case reports [1-3]. There have been no well-defined risk factors, and only a few cases of recurrence have been noted [1]. Presentation is variable and nonspecific. It can be asymptomatic or associated with other symptoms such as abdominal pain, distention, nausea, vomiting, and changes in bowel habits. The diagnosis of pneumoperitoneum is based on imaging with a plain radiograph or computed tomography.

The most common cause of pneumoperitoneum, and that which is generally of the most immediate concern, is perforated hollow viscus, as seen in over 90% of patients [2]. Other intra-abdominal causes include intestinal cystic pneumatosis, emphysematous cholecystitis, spontaneous bacterial peritonitis, or pyogenic abscess of the liver [3,4]. Intrathoracic causes include pneumothorax, trauma, bronchoperitoneal fistula, pneumomediastinum, pneumonia, ARDS, pulmonary bleb rupture, increased intrathoracic pressures (coughing, retching, and Valsalva maneuver), mechanical ventilation, or cardiopulmonary resuscitation [3,4]. Gynecologic causes include uterine rupture, pelvic inflammatory disease, aggressive sexual intercourse, and vaginal douching [3,4]. Numerous procedures can lead to pneumoperitoneum. These include endoscopic examination, paracentesis, peritoneal dialysis, peritoneal lavage, laparoscopy, laparotomy, bronchoscopy, or any pelvic instrumentation [2-4]. Post-laparoscopic pneumoperitoneum resolves within three days in 81% of patients and within seven days in 96% of patients [5]. Other rare causes that have been described include scuba diving, adenotonsillectomy, and dental extractions [2,4]. When no other etiology is identified, it is referred to as ISP.

The first step in the workup is a detailed history and physical examination. Although a plain radiograph may demonstrate pneumoperitoneum, CT is a critical tool in evaluation. CT can predict the site of GI perforation with 86% accuracy [6]. The use of upper endoscopy has also been described in the workup to evaluate for perforated viscera [7]. Endoscopy, however, has limited utility and is not able to completely evaluate the entire alimentary tract. Similarly, an upper GI contrast series can evaluate for evidence of a proximal perforation as well.

It has been stated that most cases of spontaneous pneumoperitoneum can be managed conservatively [7]. “Treat the patient, not the X-ray” [8]. This approach requires frequent abdominal examinations with close observation. Missed visceral perforation can be life-threatening and should be effectively evaluated. Conservative management should primarily be reserved for the minimally symptomatic patient with no signs of peritonitis. The nonoperative diagnosis of ISP, however, is difficult, and many such patients will undergo surgical exploration for definitive evaluation. When surgical exploration is elected, diagnostic laparoscopy is the preferred initial approach, although exploratory laparotomy may be necessary.

## Conclusions

ISP is a rare condition. This report outlines a case of recurrent ISP managed by repeated laparotomy. Knowledge of this rare phenomenon and appropriate workup may allow for the avoidance of unnecessary laparotomy. Despite this, definitively ruling out perforated hollow viscus is difficult outside of the operating room, and many patients with ISP will ultimately be taken for surgical exploration and definitive diagnosis.
